# Sitosterolemia With Two Heterozygous Variants Including a Novel Mutation c.1800T>A in the ABCG5 Gene: A Case Report of a Rare Condition in a Young Saudi Girl

**DOI:** 10.7759/cureus.63088

**Published:** 2024-06-25

**Authors:** Ali S Alquraishi, Syed Rayees

**Affiliations:** 1 Pediatric Endocrinology, Armed Forces Hospital Southern Region, Khamis Mushait, SAU; 2 Pediatrics, Armed Forces Hospital Southern Region, Khamis Mushait, SAU

**Keywords:** ezetimibe, pediatric, novel variant, abcg5 gene, sitosterolemia

## Abstract

Sitosterolemia is a rare autosomal recessively inherited lipid disorder characterized by an accumulation and deposition of phytosterols in various tissues with decreased biliary excretion leading to various complications. We report a case of a three-year-old Saudi girl who exhibited xanthomas and elevated cholesterol levels. Initially, she was misdiagnosed with familial hypercholesterolemia, but subsequent testing of the low-density lipoprotein receptor gene by next-generation sequencing ruled out this condition. Two heterozygous variants were identified in the ABCG5 gene through a whole exome sequencing study. These variants, namely c.1336C>T and c.1800T>A, have been characterized as pathogenic and likely pathogenic, respectively, with the latter being a novel mutation associated with sitosterolemia. The patient responded positively to treatment with ezetimibe, resulting in controlled cholesterol levels and decreased xanthoma size.

## Introduction

Sitosterolemia is characterized by high levels of plant sterols and low-density lipoprotein (LDL) cholesterol in the body. It is an extremely rare lipid disorder inherited by autosomal recessive mode of inheritance. The estimated prevalence is 1/1,000,000 as per the available literature on it. The recognized cause of sitosterolemia is homozygous or compound heterozygous mutations in the ABCG5 and ABCG8 genes leading to a loss of function of the adenosine triphosphate (ATP)-binding cassette [1]. ABCG5 and ABCG8 are responsible for synthesizing specific proteins in the small intestine and liver cells, essential for transporting plant sterols to the human intestine and bile ducts. Sitosterolemia occurs when there is a dysfunction in transporting these sterols from the intestinal epithelium to the lumen and from the liver to the bile [2]. This disease exhibits a broad spectrum of clinical features, ranging from asymptomatic cases to severe, potentially fatal conditions. Symptoms commonly include skin xanthoma, atherosclerosis, arthritis, hepatomegaly, splenomegaly, lipid-type red blood cells, large platelets, and thrombocytopenia. Elevated levels of total blood cholesterol and LDL cholesterol are often detected in affected individuals. The complete clinical picture of this disease may be underestimated largely due to underdiagnosis [3]. Several cases of sitosterolemia are still being misdiagnosed as familial hypercholesterolemia, leading to an inaccurate estimation of the disorder's prevalence [4]. Children may exhibit planar, tuberous, or tubero-eruptive xanthomas in different parts of their bodies. If buttock xanthomas are present, it should raise suspicion for this disorder. A genetic study can be conducted to analyze DNA mutations in the ABCG5 or ABCG8 gene and confirm the diagnosis of sitosterolemia [5]. It is recommended to follow a diet low in plant sterols and consider treatment with ezetimibe [6].

## Case presentation

We present a case study involving a three-year-old girl of Arab ethnicity who was born to non-consanguineous parents. The child was delivered at term through spontaneous vaginal delivery and did not require admission to the neonatal intensive care unit. Notably, her father, grandfather, and grandmother have hypercholesterolemia. She has six siblings who are unaffected and in good health. At the age of two and a half years, the child's parents noticed the presence of yellowish skin lesions at various sites on her body. Concerned about these findings, they sought medical advice at a hospital, where it was determined that she had elevated cholesterol levels. Consequently, they brought her to our hospital for further monitoring and care. She was evaluated in the endocrinology clinic for appropriate assessment and treatment. Upon physical examination, no dysmorphic traits were evident, and the patient was developmentally normal for her age. Xanthomas were observed bilaterally in the axilla, hands' dorsum, knees, and sacral area. No xanthelasma was noted. The chest examination revealed clear breath sounds, a normal cardiovascular system with regular heart rhythms, and a soft, non-tender abdomen.

Initial investigations (Table 1) included a complete blood count, which yielded normal results and peripheral smear showed occasional large platelets. However, the lipid profile revealed elevated cholesterol levels and LDL levels. Alternatively, a range of other tests, such as liver function, coagulation profile, bone profile, thyroid function, glycated hemoglobin, creatinine kinase, and C-reactive protein, all demonstrated normal values. Additionally, the echocardiogram showed no abnormalities.

**Table 1:** Laboratory values before and after treatment

Measured entity	Value at presentation	Value post-treatment with ezetimibe	Reference ranges
White blood cells	6.80 x 10^9/L	5.4 x 10^9/L	4.5-13.5 x 10^9/L
Hemoglobin	12 g/dL	13 g/dL	11-15 g/dL
Platelets	300 x 10^9/L	230 x 10^9/L	150-400 x 10^9/L
Cholesterol	17 mmol/L	4.56 mmol/L	2.14-5.45 mmol/L
Triglycerides	0.74 mmol/L	0.87 mmol/L	0.45-1.71 mmol/L
Low-density lipoprotein (LDL)	8.2 mmol/L	3 mmol/L	1.30- 1.81 mmol/L
High-density lipoprotein (HDL)	0.64 mmol/L	1 mmol/L	1.29-1.53 mmol/L

Due to a strong family history of familial hypercholesterolemia, a sample was taken for genetic study of the low-density lipoprotein receptor (LDLR) gene. A consultation with a dietician was sought for dietary modifications. During follow-up it was observed that the patient was not compliant with dietary modifications, the xanthomas were increasing in size and the cholesterol levels remained high. Molecular genetic analysis of the LDLR gene using next-generation sequencing (NGS) yielded negative results. To explore other potential causes of hypercholesterolemia and xanthoma, a whole exome sequencing (WES) study was conducted (Table 2). This study identified two heterozygous variants in the ABCG5 gene, specifically c.1336C>T and a novel variant c.1800T>A, confirming the diagnosis of sitosterolemia.

**Table 2:** Whole exome sequencing study MIM: Mendelian Inheritance in Man, AR: Autosomal Recessive, gnomAD: genome Aggregation Database, MAF: Minor Allele Frequency

Gene (isoform)	Phenotype MIM Number (Mode if Inheritance)	Variant	Zygosity	MAF gnomAD (%)	Classification
ABCG5 (NM_022436.3)	618666 (AR)	c.1336C>T p.(Arg446*)	heterozygous	0.022	pathogenic
		c.1800T>A p.(Cys600*)	heterozygous	0	Likely pathogenic

**Figure 1:**
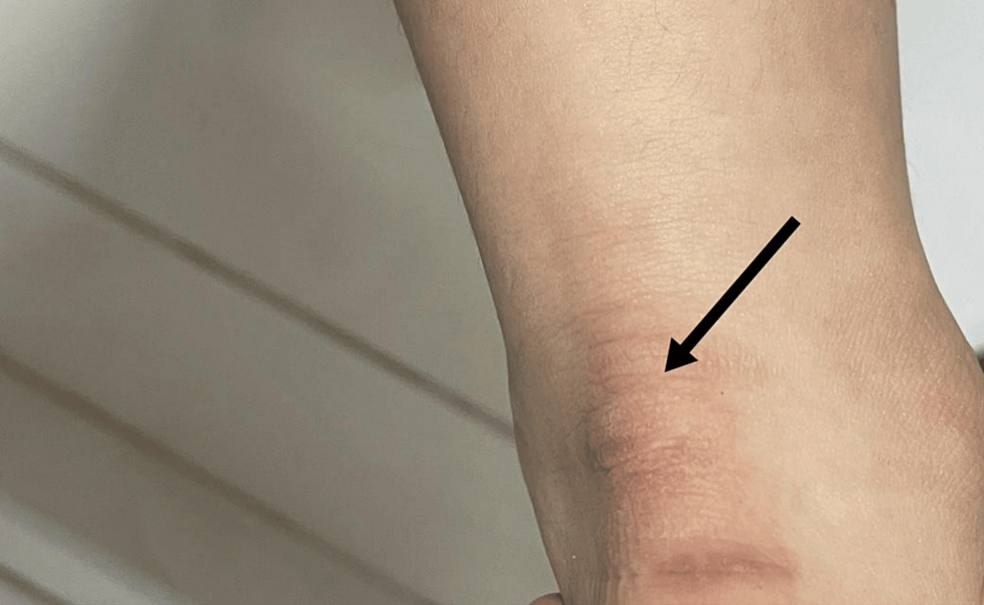
Healed tendinous xanthoma at the achilles tendon post treatment with ezetimibe

**Figure 2:**
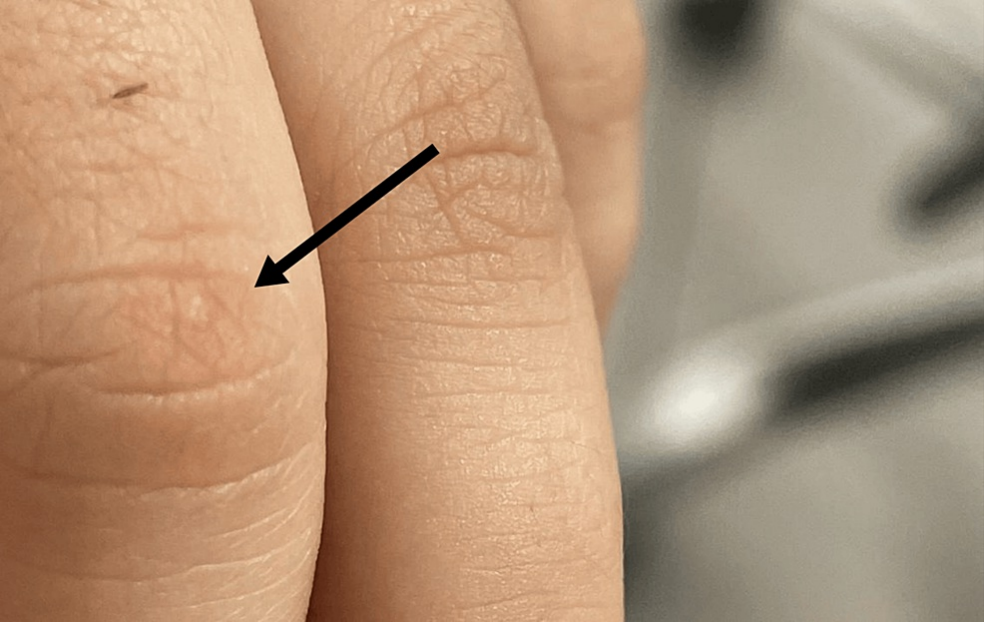
Healed finger xanthoma post treatment

**Figure 3:**
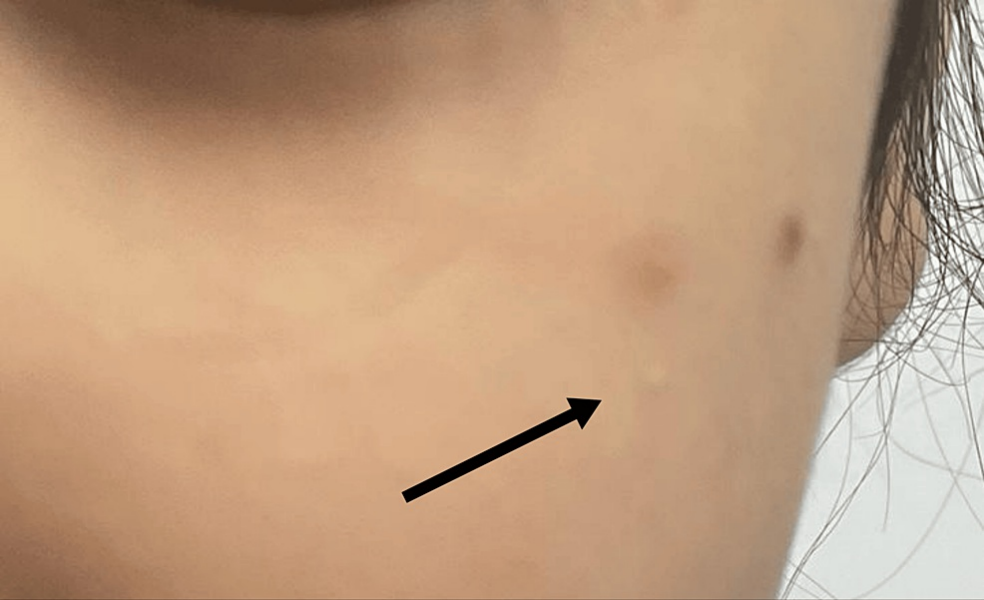
Xanthoma post treatment reduced in size

These findings, in conjunction with the confirmed diagnosis of sitosterolemia, led to the initiation of treatment with ezetimibe 10 mg orally once daily. It is worth noting that this medication is not approved for use in the pediatric age. However, due to the high risk of myocardial infarction and cerebral vascular accident associated with this disease, as well as the absence of alternative treatments, the parents were informed and consented to the use of ezetimibe. The diet was modified to avoid plant sterols in food. After two weeks of starting treatment, the patient developed a skin rash on both arms, which was determined to be a mild drug reaction as per the dermatologist. Consequently, the dosage of ezetimibe was tapered to half. Throughout the course of clinic follow-up, there was significant improvement in the patient's cholesterol levels and xanthoma (Figures 1-3). Currently, the patient is doing well on a daily dose of ezetimibe 2.5 mg, with regular follow-up appointments in the endocrinology, cardiology, and dermatology clinics.

## Discussion

Sitosterolemia is a rare inherited lipid metabolism disorder with less than 400 reported cases worldwide. It is primarily caused by autosomal recessively inherited mutations in the ABCG5 and ABCG8 genes located on chromosome 2p21. Individuals harboring homozygous or compound heterozygous mutations in these genes exhibit increased absorption of dietary fats coupled with decreased excretion through the biliary system as they affect the proteins called sterolin-1 and sterolin-2, which combine to form the adenosine triphosphate binding cassette sterol excretion transporter leading to highly elevated levels of plant sterols and LDL cholesterol in the bloodstream. The subsequent accumulation of phytosterols in various tissues, particularly in the vasculature, contributes to the development of atherosclerosis. Moreover, a significant proportion of sitosterolemia patients experience hematologic abnormalities such as stomatocytosis, large platelets, and hemolytic anemia, which can be attributed to the harmful effects of excessive phytosterols on red blood cells and platelet formation and function. Individuals diagnosed with sitosterolemia often exhibit elevated cholesterol levels, which can be mistakenly identified as familial hypercholesterolemia, resulting in improper treatment [1,2]. Among Asian patients, ABCG5 mutations are found in nearly all cases, while ABCG8 mutations are more commonly detected in Caucasian patients. Although adult patients with sitosterolemia typically exhibit normal levels of plasma cholesterol, pediatric patients may present with severe hypercholesterolemia. The clinical presentation of sitosterolemia is highly diverse, ranging from patients showing no symptoms to those experiencing severe, life-threatening cases. The common manifestations associated with this condition are arthralgia, xanthomas on extensor surfaces, anemia, stomatocytosis, macrothrombocytopenia, thrombocytosis, elevated transaminases, hepatomegaly, splenomegaly, premature atherosclerosis and severe hypercholesterolemia in children. The complete clinical spectrum of sitosterolemia may not be fully understood due to a lack of proper diagnosis [3].

Diagnosis of this condition involves measuring plant sterol levels in plasma through gas chromatography-mass spectrometry (GC-MS). Traditional enzymatic cholesterol testing cannot differentiate between plant sterols and cholesterol, potentially resulting in inaccurately high total cholesterol readings. Mutation analysis can be conducted by sequencing the ABCG5 and ABCG8 genes.

It is crucial to distinguish between sitosterolemia and familial hypercholesterolemia, as traditional cholesterol-lowering diets high in vegetable oils are not recommended for sitosterolemia patients. Dietary interventions for sitosterolemia focus on reducing phytosterol buildup in the body [4,5]. Reduction in dietary sterol intake has proven to be effective in treating hyperlipidemia in sitosterolemia, as opposed to statin treatment which often yields poor results. The absence of improvement with statin treatment could serve as a diagnostic indicator. Studies have demonstrated that ezetimibe can effectively lower levels of total cholesterol and plant sterols in individuals with sitosterolemia [6]. Therapy primarily involves restricting the intake of cholesterol and plant sterols found in various food sources such as vegetable oils, margarine, nuts, seeds, avocado, and chocolate. Additionally, the use of sterol absorption inhibitors like ezetimibe or bile acid sequestrants is recommended. It is important to note that certain shellfish like clams, oysters, and scallops also contain shellfish sterols that are hyper-absorbed and should be avoided. While cholesterol restriction is not advised for children under two years old and pharmacotherapy is generally not recommended for those under 10 years old, exceptions may be made for individuals with exceptionally high cholesterol levels. Studies have shown that a low-fat/low-cholesterol diet can be safe and effective for infants and children [7]. Ezetimibe exerts its therapeutic effects by selectively targeting the intestinal NPC1L1 transporter, thereby hindering the absorption of sterols. Consequently, this action reduces plasma plant sterol levels, ameliorates hematological abnormalities, and successfully reduces xanthomas [8,9].

## Conclusions

In cases of sitosterolemia, a rare inherited lipid disorder, it is imperative to explore a differential diagnosis when children present with severe hypercholesterolemia, with a probable diagnosis of familial hypercholesterolemia. Our case exemplifies the common misdiagnosis of sitosterolemia, where a patient with a family history of familial hypercholesterolemia was initially diagnosed and treated as familial hypercholesterolemia. The early introduction of ezetimibe treatment and a low plant sterol diet following a confirmed diagnosis has enhanced patient outcomes. Where available, early genetic analysis and phytosterol measurements are essential to promptly initiate treatment and prevent various complications. It is crucial to promptly diagnose and closely monitor cardiovascular events in pediatric populations as there have been reports of atherosclerosis and myocardial infarction at a young age. Genetic counseling for family members is crucial, and regular follow-up is advised to monitor for potential complications.
